# Atrial lead perforation early after device implantation—a case report and literature review

**DOI:** 10.3389/fsurg.2024.1290574

**Published:** 2024-04-05

**Authors:** Zefeng Wang, Zhongyu Yuan, Haiwei Li, Ke Zhang, Hongkai Zhang, Xiaoyan Li, Changhua Wang, Yilun Tian, Yiqing Shen, Xiaoping Zhang, Yongquan Wu

**Affiliations:** ^1^ Department of Cardiology, Beijing Anzhen Hospital, Capital Medical University, Beijing, China; ^2^ Cardiovascular Surgery Center, Beijing Anzhen Hospital, Capital Medical University, Beijing, China; ^3^ Department of Radiology, Beijing Anzhen Hospital, Capital Medical University, Beijing, China; ^4^ Beijing Anzhen Hospital, Capital Medical University, The Key Laboratory of Remodelling-Related Cardiovascular Diseases, Ministry of Education, Beijing Institute of Heart, Lung and Blood Vessel Diseases, Beijing, China

**Keywords:** pacemaker complications, atrial perforation, active fixation, cardiac perforation, active fixation lead

## Abstract

We report three patients with screw-in lead perforation in the right atrial free wall not long after device implantation. All the patients complained of intermittent stabbing chest pain associated with deep breathing during the implantation. The “dry” epicardial puncture was utilized to avoid hemopericardium during lead extraction in the first case. The atrial electrode was repositioned in all cases and replaced by a new passive fixation lead in two patients with resolution of the pneumothorax or pericardial effusion. A literature review of 50 reported cases of atrial lead perforation was added to the findings in our case report.

## Background

Lead perforations associated with the implantation of cardiovascular electronic devices are rare, with published rates of 0.1%–0.8% for permanent pacemakers and 0.14%–5.2% for implantable cardioverter defibrillators (ICDs) (1). The majority of cases occurred during implantation or within the first 24 h post procedure, but numerous instances of lead perforation occurring within days or months after procedure have also been reported (2). A wide range of typical and atypical symptoms can be observed, including pericardiac or pleuritic chest pain, hiccups, dyspnea, shortness of breath, hemoptysis, and even hemodynamic instability. Postoperative changes in electrical parameters may be a clue in asymptomatic patients (3). Transthoracic echocardiography (TTE) may reveal a hemodynamically relevant pericardial effusion, and the perforation site may be confirmed by computed tomography (CT). Detection of pneumothorax or hematopneumothorax on chest radiography (CXR) could also have auxiliary diagnostic value (2, 3). Furthermore, acute progression to cardiac tamponade is probably the most catastrophic consequence of a lead perforation, hence rapid management is crucial. Multiple studies in the literature have reported ventricular lead perforations induced by active, ICD, or even passive electrodes, regardless of the duration after the procedure. However, atrial lead perforations were less mentioned and mainly consisted of case reports. Herein we aimed to analyze the underlying mechanism of atrial lead perforation in the aspect of anatomy and provide the readers with some lessons learned for the diagnosis and treatment strategies of atrial perforation through this case series combined with a literature review.

## Case presentation

### Case 1: atrial lead perforation with right-sided hemothorax

**Figure 1:**
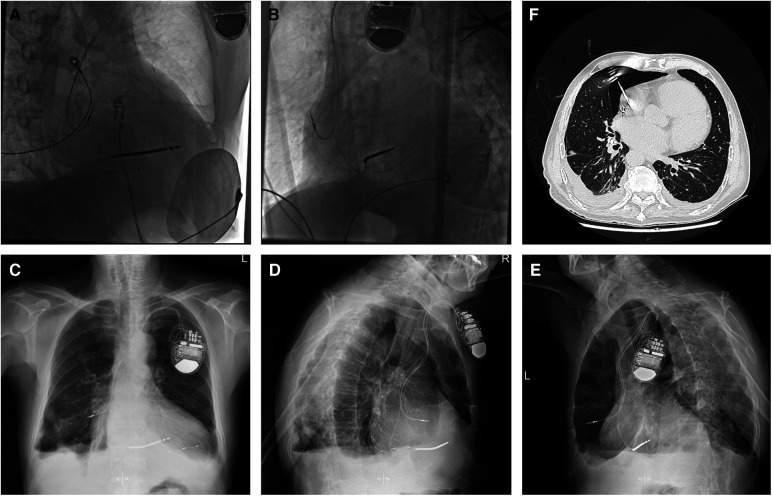
Fluoroscopy images of the initial active fixation lead in RA free wall of case 1 [(**A**) RAO view and (**B**) LAO view]. Imaging examinations of case 1 obtained on readmission (**C–F**). Chest radiograph indicating the atrial lead protruding outside the contours of RA [(**C**) AP view, (**D**) RL view, and (**E**) LL view]. Chest CT scan showing the tip of the atrial electrode touching the right upper lobe of the lung and causing pneumothorax (**F**). AP, anterior posterior; CT, computed tomography; LAO, left anterior oblique; LL, left lateral; RA, right atrium; RAO, right anterior oblique; RL, right lateral.

A 71-year-old male patient was readmitted to our hospital on a complaint of right thoracic stabbing pain with alerting of a previously implanted cardiac resynchronization therapy defibrillator. He had non-ischemic dilated cardiomyopathy (DCM), left ventricular (LV) ejection fraction of 20%, left bundle branch block with a QRS duration (QRSd) of 175 ms, and New York Heart Association Class III symptoms despite optimal medical treatment. The procedure was then performed via the left axillary vein with a bipolar active fixation lead (Medtronic, CapSureFix Novus 5076, Minneapolis, MN, USA) positioned in the right atrial (RA) free wall (Figures 1A,B), a single coil lead (Medtronic, Sprint Quatro 6935M) screwed in the right ventricular (RV) anterior-mid septum, and a quadripolar lead (Medtronic, Attain Performa 4298) placed in the left ventricular anterior lateral wall successfully. At the time of implantation, the pacing threshold of the atrial lead was 0.8 V at 0.42 ms, the P-wave amplitude was 3 mV, and the impedance was 560 Ω. The pacing threshold of the shock lead was 0.9 V at 0.42 ms, the R-wave amplitude was 9 mV, and the impedance was 580 Ω. The left ventricular lead pacing threshold was 0.8 V without phrenic nerve stimulation. The postoperative electrocardiogram (ECG) showed a narrowed QRSd of 107 ms. The patient was discharged free of symptoms 2 days later on beta-receptor blocker, angiotensin receptor blocker, and diuretic medication.

On readmission, his vital signs were stable and within the normal range except for oxygen saturation of 93% in the room air. The physical examination revealed decreased respiratory sounds on the right side of the chest. Laboratory tests, including high-sensitive troponin T and serum myocardial enzyme levels, were within normal limits, and the ECG showed no ST-T changes or new Q wave, but a reprolonged QRSd of 203 ms excluded myocardial ischemia. Device interrogation revealed undersensing and loss of capture at maximal pacing output of the atrial lead. CXR showed an enlarged cardiac silhouette with the atrial lead protruding outside the contours of the RA (Figures 1C–E). However, CT visualized the tip of the helix of the atrial screw-in electrode touching the right upper lobe of the lung and causing pneumothorax (Figure 1F). As the TTE did not show any pericardial effusion, we considered that it was a pneumothorax that caused the chest pain.

**Figure 2:**
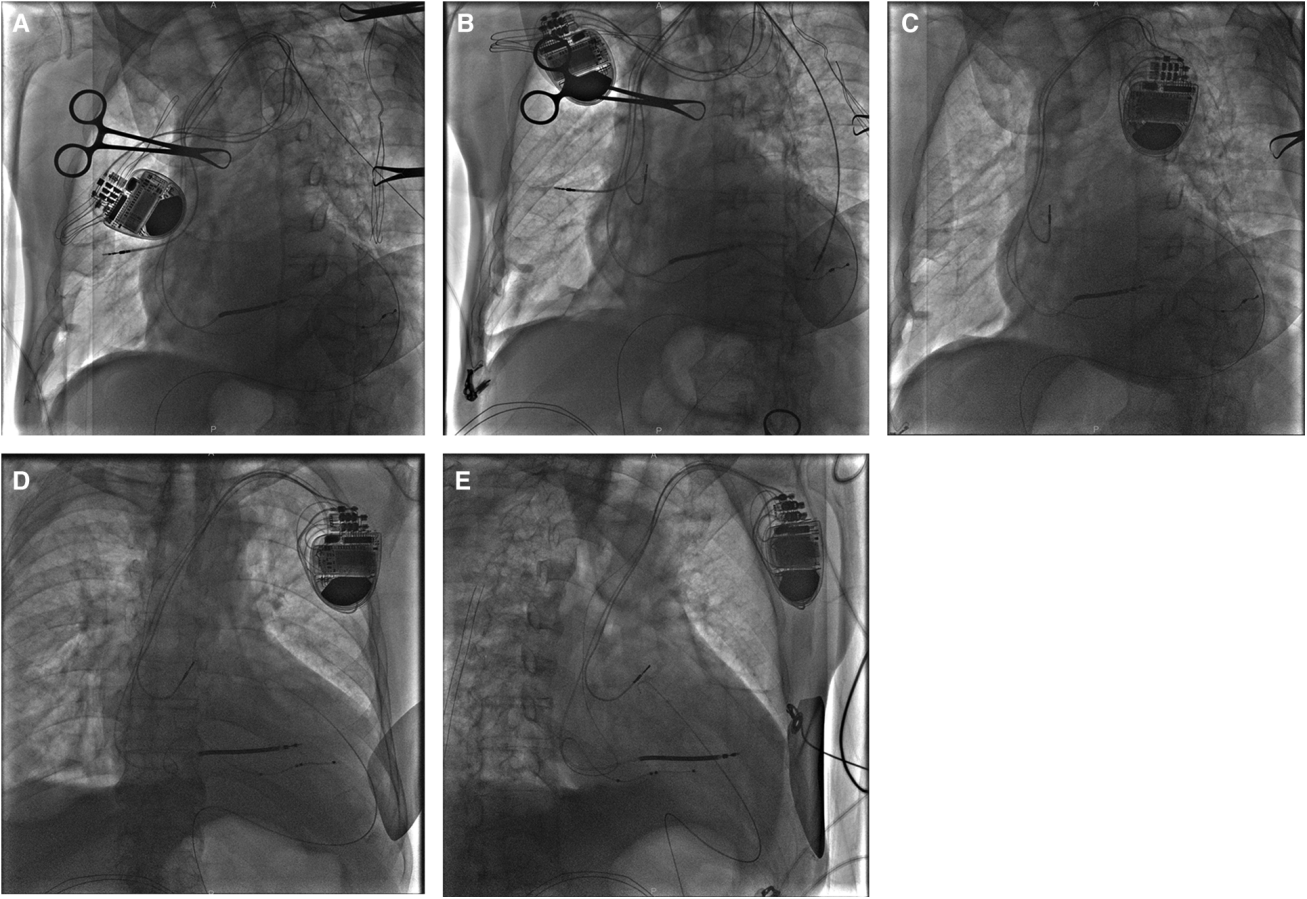
In case 1, a J-tipped guidewire was placed into the pericardial space using an LAO view (**A**) and was left *in situ* in case of future cardiac effusion caused by electrode extraction. A new passive fixation lead was implanted in proximal RAA in the LAO view (**B**). After complete retraction of the target atrial lead, fluoroscopy visualized no pericardial effusion and the left ventricular lead maintaining in the appropriate position was found in different views [(**C**) LAO view, (**D**) AP view, and (**E**), RAO view]. AP, anterior posterior; LAO, left anterior oblique; RAA, right atrial appendage; RAO, right anterior oblique.

Given that the helix tip of the protruding atrial lead bouncing with the heartbeat would probably cause further damage to the lung tissue, and that the patient was hemodynamically stable without a tendency to cardiac tamponade inclination, retention of the original atrial electrode or mini-thoracotomy was not considered as an optimal option. With ECG, blood pressure, oxygen saturation monitoring, prophylactic antibiotic therapy, and the pacemaker programmed for VVI ventricular sensing tracking pacing mode, the “dry” epicardial puncture was performed (4). The needle tip was initially directed to the middle point between the ventricular apex and annulus in the right anterior oblique (RAO) fluoroscopic projection, while the anterior–posterior angle was adjusted under the left lateral view. The J-tipped guidewire was placed through the needle into the pericardial space using a left anterior oblique (LAO) view (Figure 2A) and was left *in situ* in case of future cardiac effusion caused by electrode extraction. A new tined lead (Medtronic, CapSure Sense 4574) was implanted in the proximal right atrial appendage (RAA) (Figure 2B). The gentle traction of the target atrial lead was carefully applied using a LAO view under TTE monitoring, with an urgent thoracotomy planned in the event of retraction failure. Fluoroscopy visualized no pericardial effusion and the left ventricular lead remained in the appropriate position at various angles (Figures 2C–E). After 30 min of observation, TTE showed no pericardial effusion in the subxiphoid view. No complications occurred during or after the operative procedure. Iterative bedside TTEs during the postoperative 48 h showed no pericardial effusion and the pacing parameters were satisfactory, therefore the residual J-tipped guidewire was extracted. CXR showed spontaneous resolution of the pneumothorax. The patient was discharged after 7 days with a narrowed QRSd of 118 ms. During the 3-month follow-up, the patient remained asymptomatic and continued to show satisfactory device readings.

### Case 2: atrial lead perforation with pericardial effusion

**Figure 3:**
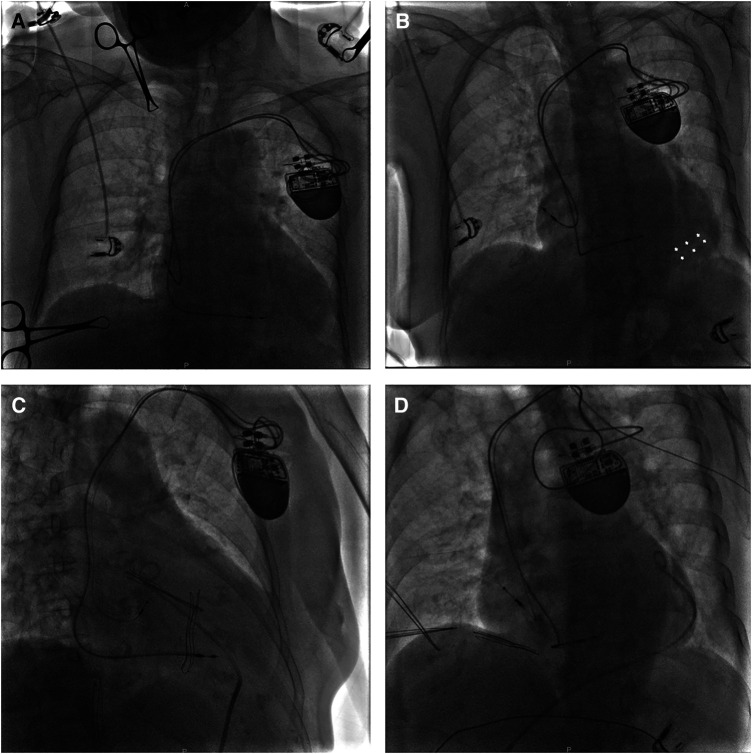
A fluoroscopy image of case 2 showing the initial active fixation lead in RA free wall [(**A**) AP view]. A bright band at the bottom of the heart suggesting pericardial effusion in the LAO view [(**B**) white arrows]. After relief of chest pain by drainage through a pigtail catheter, a new passive fixation lead was placed in the distal RAA [(**C**) LAO view and (**D**) RAO view]. AP, anterior posterior; LAO, left anterior oblique; RA, right atrium; RAA, right atrial appendage; RAO, right anterior oblique.

A 78-year-old scrawny female patient with a symptomatic sick sinus syndrome (SSS) and amaurosis fugax was scheduled for elective implantation of a dual-chamber pacemaker implantation. Despite a history of paroxysmal atrial fibrillation, the patient was not regularly taking oral anticoagulants. A screw-in atrial lead (Boston Scientific, Ingevity 7741, North Charleston, SC, USA) was inserted from the left axillary vein and attached to the RA free wall (Figure 3A). Similarly, a screw-in ventricular lead (Boston Scientific, Ingevity 7742) was positioned in the RV septum. The pacing threshold of atrial lead was 0.8 V at 0.48 ms, the P-wave amplitude was 4.6 mV, and the impedance was 435 Ω. The ventricular lead pacing threshold was 0.9 V at 0.48 ms, the R-wave amplitude was 8.6 mV, and the impedance was 622 Ω.

A few minutes after being transferred from the operating table to the flat car, the patient began to complain of continuous left-sided chest pain with nausea. Of note, her blood pressure suddenly dropped to 100/60 mmHg. Immediate fluoroscopy demonstrated weakened cardiac pulsation and moderate pericardial effusion (Figure 3B). Pacemaker interrogation revealed loss of both atrial sensing and capture. With intravenous infusion of dopamine to improve the blood pressure to 105/62 mmHg and 0.9% sodium chloride for rapid rehydration and hemodynamic stability, x-ray guided pericardiocentesis was performed in the catheterization (cath) lab under local anesthesia. After drainage of incoagulable blood through a pigtail catheter, the patient reported remission of chest discomfort, and fluoroscopy visualized rehabilitative cardiac impulse and pericardial effusion much less than before. During a 30-min observation, her blood pressure fluctuated around 125/70 mmHg. A new tined lead (Boston Scientific, Fineline II Sterox 4480) was then placed in the distal RAA (Figures 3C,D) with pacing threshold of 1 V, lead impedance of 598 Ω*,* and P-wave amplitude of 2.6 mV. Reiterant bedside TTEs revealed no pericardial effusion and the patient was discharged 3 days after the procedure. The patient made a full recovery with no procedure-related complications at the 18-month follow-up.

### Case 3: atrial lead perforation with pericardial effusion

**Figure 4:**
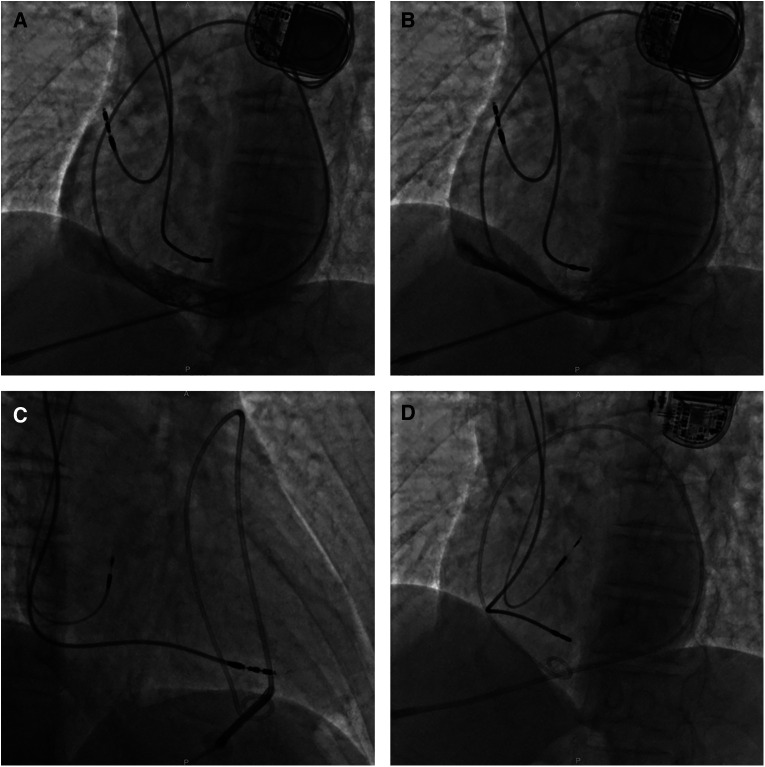
A fluoroscopy image of case 3 showing initial active fixation lead in lateral RA and moderate pericardial effusion [(**A**) LAO view]. X-ray guided pericardiocentesis was performed using an LAO view and a pigtail catheter was inserted into the epidural space for continuous drainage, thus hemodynamic stability was obtained (**B**). Then a new active fixation electrode was implanted in RA septum [(**C**) RAO view and (**D**) LAO view]. LAO, left anterior oblique; RA, right atrium; RAO, right anterior oblique.

A 60-year-old underweight female patient presented with symptomatic SSS and multiple syncope, which led to an uncomplicated implantation of a dual-chamber pacemaker. The ECG Holter showed Mobitz type II second-degree sinoatrial block with accidental junctional escapes and atrial premature beats. Implantation was uneventful with both leads inserted via the left axillary vein. The atrial lead (Biotronik, Solia S 53, Berlin, Germany) was actively fixed in the RA free wall (Figure 4A) and the ventricular lead (Biotronik, Solia S 60) was fixed in the RV septum. The atrial lead pacing threshold was 2 V at 0.48 ms, the P-wave amplitude was 3.3 mV, and the impedance was 532 Ω. The pacing threshold of the ventricular lead was 1.5 V at 0.48 ms, R-wave amplitude was 12.5 mV, and the impedance was 757 Ω.

Approximately 30 min after returning to the ward, the patient complained of severe breath-related left-sided pain with nausea and emesis. Pacemaker interrogation revealed the failure of atrial pacing and sensing and decreased lead impedance. Blood pressure monitoring revealed a sudden drop to 70/40 mmHg and distant heart sounds were auscultated. An intravenous infusion of 0.9% sodium chloride 500 ml for rapid rehydration and an intravenous injection of dopamine to improve hemodynamics were immediately administered. With a heart rate of 76 bpm, atropine was also injected intravenously, followed by continuous dopamine infusion at 8 μg/kg/min, with blood pressure maintained at 100/50 mmHg and heart rate at 68 bpm. An emergency bedside TTE defined a mild to moderate pericardial effusion (anterior 9 mm, posterior 4 mm, and RV free wall 12 mm), confirming the suspicion of atrial lead perforation. X-ray guided pericardiocentesis was performed and a pigtail catheter was inserted into the epidural space for continuous drainage of 400 ml of incoagulable blood (Figure 4B). The patient reported significant amelioration of chest pain. Afterward, the atrial lead was removed transvenously and a new screw-in lead (Medtronic, Select-Secure 3830) was implanted in the RA septum (Figures 4C,D) with a pacing threshold of 0.8 V at 0.48 ms, P-wave amplitude of 3.1 mV, and impedance of 783 Ω. The patient was observed for 6 days in the ward postoperatively. The patient had no recurrent pericardial effusion during 6 months of clinical and echocardiographic follow-up, and pacemaker interrogation was unremarkable.

### Review of the literature

We searched PUBMED for articles on atrial lead perforations from the period between 1986 and 2023. The inclusion criterion was myocardial or vascular injury due to atrial lead dislocation. The exclusion criteria were as follows: perforation caused by (i) sheath or catheter intraoperatively, (ii) erosive ventricular lead, (iii) lead extraction, and (iv) the piercing site not verified by any imaging examination or direct visualization during thoracotomy. As a result, 50 patients, including our patients, were enrolled (Table 1). There were 21 men and 29 women, ranging in age from 23 to 88 years (average 66.8 ± 14.0).

**Table 1:** Published cases of atrial lead perforation between 1986 and 2021.

Author	Gender, age	Lead	Position	Perforation	Pace/sense	Delay	Symptoms	Imaging	Management
Sussman Chiles (5)	F, 80	Tined lead	Not reported	Pleura	Not reported	4 months	Chest pain, hiccups, syncope	TTE, CXR, CT	Thoracentesis, thoracotomy (lead extraction, RA repair, epicardial lead implantation)
Irwin (6)	F, 80	Tined lead	Not reported	Pleura	No capture	25 weeks	Syncope, hiccups	CXR, CT	Thoracentesis, thoracotomy (lead extraction, epicardial electrode implantation, RA repair)
Ho (7)	F, 79	Screw-in lead	Not reported	Pleura	Intermittent no sensing	4 h	Dyspnea	CXR	Thoracentesis, lead extraction, tined lead implantation
Aizawa (8)	F, 81	Screw-in lead	RA free wall	Pericard	Undersensing	2 days	Chest pain	TTE, CT	Thoracotomy, lead extraction, tined lead implantation
Tran (9)	M, 33	Screw-in lead	Not reported	Pleura	Not reported	4 days	Pleuritic chest pain	CXR	Thoracotomy (pericardium repair)
Dilling-Boer (10)	M, 51	Screw-in lead	RAA	Pleura	Undersensing, elevated threshold	3 days	Pericarditic chest pain	CXR, CT, TTE	Thoracentesis, pericardiotomy, lead reposition
Velavan (11)	F, 64	Screw-in lead	Lateral RA	Pericard	Not reported	1 month	Back pain, hemodynamic unstable	TTE	Thoracotomy (pericardiotomy, RA repair)
Howell (12)	F, 49	Not reported	Not reported	Pericard	Elevated threshold	3 months	Nausea, decreased appetite, abdominal bloating, dyspnea	CT, CXR, TTE	Pericardiocentesis, lead reposition
Henrikson (13)	F, 66	Screw-in lead	Not reported	Pericard	Undersensing	2 weeks	Asymptomatic	CT	Device reprogramming to VVIR
Geyfman (14)	F, 50	Screw-in lead	RA free wall	Pericard	No effect	2 days	hemodynamic unstable	TTE	Pericardiocentesis, thoracotomy (RA repair)
Geyfman (14)	F, 56	Screw-in lead	Lateral RA	Pericard	No effect	6 h	Chest discomfort, collapse	TTE	Pericardiocentesis, thoracotomy (RA repair)
Geyfman (14)	F, 72	Screw-in lead	RA free wall	Pericard	No effect	1 week	Chest pain, hemodynamic unstable	CXR, TTE	Pericardiocentesis, lead repositioned to RAA
Spencker (15)	F, 56	Not reported	Not reported	Pleura	No capture, increased impedance	4 weeks	Asymptomatic	CT	Lead repositioned to RAA
Namazi (16)	M, 48	Screw-in lead	Lateral RA	Pleura	No capture	2 weeks	Hemoptysis	CXR, CT	Lead reposition
Sadamatsu (17)	F, 63	Screw-in lead	RAA	Pericard	Intermittent capture	5 years	Asymptomatic	CXR, CT	Thoracotomy (lead removal)
Schiariti (18)	M, 23	Screw-in lead	RA free wall	Pericard	No effect	4 months	Chest pain, hemodynamic unstable	TTE	Pericardiocentesis, lead extraction, tined lead implantation
Peddi (19)	F, 77	Screw-in lead	Not reported	Pericard	Not reported	3 days	Chest pain	CT, TTE	Thoracotomy (pericardiotomy, RA repair)
Antonelli (20)	M, 74	Screw-in lead	Anterior RA	Pericard	Not reported	2 h	Chest pain	TTE	Thoracotomy (pericardiotomy, lead removal, RA repair)
Hussain (21)	F, 75	Not reported	RA free wall	Pericard	Undersensing, no capture	1 week	Asymptomatic	CXR, CT	Lead repositioned to RAA, RA repair via the da Vinci robotic system
Khoueiry (22)	M, 67	Tined lead	RAA	RCA	Elevated threshold	53 months	Asymptomatic	Coronary angiography, CT	Thoracotomy (lead resection, RCA ligation, RA repair)
Nakanishi (23)	M, 66	Screw-in lead	RAA	Pericard	No effect	7 years	Not reported	None	Pericardiocentesis, thoracotomy (RAA repair)
O’Neill (24)	F, 65	Not reported	Not reported	Pleura	Not reported	2 years	Pleuritic chest pain, shortness of breath, nausea, emesis, shock	CXR, CT	Pericardiocentesis, thoracotomy (lead removal, RA repair)
Souretis (25)	M, 65	Screw-in lead	Not reported	Pericard	Increased impedance, no capture	>1 month	Dyspnea, hemodynamic unstable	CXR, TTE	Thoracotomy (pericardiotomy, RAA repair)
Higny (26)	M, 66	Screw-in lead	RA free wall	Pleura	Undersensing, no capture	5 days	dry cough, retrosternal and pleuritic chest pain	CT	Lead extraction, new screw-in lead implanted at RAA
Di Marco (27)	M, 54	Screw-in lead	Not reported	Aorta, Pericard	No effect	6 h	Chest pain, dizziness, hemodynamic unstable	TTE	Thoracotomy (pericardiotomy, aorta and RA repair)
Rath (28)	F, 76	Not reported	Not reported	SVC	No effect	2 h	Hemodynamic unstable	CXR, CT	Thoracotomy (pericardiotomy, lead repositioned to RAA)
De Schryver (29)	M, 33	Screw-in lead	RAA	Pleura	No sensing and capture	8 months	Asymptomatic	CXR, CT	Lead removal
Baird (30)	M, 75	Screw-in lead	RAA	Pleura	No effect	6 months	Chest pain, shortness of breath	CT	Conservative
Nakagawa (31)	F, 72	Screw-in lead	RAA	RCA	Not reported	3.5 h	Nausea, hemodynamic unstable	TTE	Pericardiocentesis, thoracotomy (RCA repair)
Rali (32)	F, 73	Screw-in lead	Not reported	Pleura	No effect	Intraoperatively	Nausea, emesis, dyspnea	CXR	Thoracentesis
van Gelder (2)	M, 61	Tined lead	Not reported	Pleura	No capture	9 years	Asymptomatic	Not reported	Thoracoscope (lead resection), lead removal
Ishizue (33)	M, 67	Screw-in lead	Anterolateral RA	Pleura	No effect	4 days	Asymptomatic	CXR, CT	Conservative
Saradna (34)	M, 88	Screw-in lead	Not reported	Pleura	Intermittent capture	12 days	Chest pain	CXR, TTE	Lead reposition
Aktaa (35)	F, 76	Screw-in lead	Not reported	Pleura	No effect	5 days	Pleuritic chest pain	CTPA	Lead extraction, tined lead implantation
Schreiber (36)	F, 87	Screw-in lead	Lateral RA	Pleura	No effect	3 years	Hemoptysis	CT	Bronchoscopy, lead extraction, new screw-in lead implantation
Shenthar (37)	M, 68	Screw-in lead	Not reported	Pericard	No effect	1 year	Shortness of breath	TTE, CT	Pericardiocentesis, lead reposition
Zhou (38)	F, 61	Screw-in lead	Not reported	Pleura	No sensing and capture	2 months	Chest pain, hemoptysis	CXR, CT	Lead extraction, new screw-in lead implanted at RAA
Futami (39)	F, 71	Screw-in lead	Anterolateral RA	Pleura	Elevated threshold	2 weeks	Chest pain, dyspnea	CXR, CT, TTE	Thoracentesis, lead removal
Gianni (40)	F, 53	Screw-in lead	Not reported	Pleura	Undersensing, no capture	2 weeks	Chest pain, shortness of breath	CXR, CT	Lead reposition (high RAA), mini-thoracotomy (RA repair)
Olesen (41)	F, 77	Screw-in lead	Lateral RA	Pleura	No effect	Shortly	Asymptomatic	CXR	High flow oxygen, lead repositioned to RAA
Grebmer (42)	M, 82	Screw-in lead	Anterolateral RAA	Pericard	Varying sensing	5 min	Hemodynamic unstable	TTE	Pericardiocentesis, thoracotomy (lead extraction, epicardial electrode implantation, RA repair)
Grebmer (42)	M, 84	Screw-in lead	Lateral RA	Pleura	No effect	5 days	Pleuritic chest pain	CT	Lead removal, thoracentesis
Grebmer (42)	F, 63	Screw-in lead	Lateral RA	Pleura	No effect	Immediately	Pleuritic chest pain	CXR	Lead repositioned to RAA
Grebmer (42)	M, 82	Screw-in lead	Lateral RA	Pericard	Not reported	Immediately	Pericarditic chest pain	TTE, CT	Pericardiocentesis, lead removal
Tsuji (43)	M, 74	Screw-in lead	RAA	RCA	No effect	7 h	Hemodynamic unstable	TTE	Pericardiocentesis, thoracotomy (RCA repair)
Yilancioglu (44)	F, 65	Not reported	Not reported	Pleura	Elevated threshold, no sensing	3 days	Right upper quadrant pain	CXR, CT	Lead repositioned to RAA
Enokizono (45)	F, 82	Screw-in lead	RAA	Pleura	No effect	6 years	Chest pain	CT	Conservative
Wang (this article)	M, 71	Screw-in lead	RA free wall	Pleura	Undersensing, no capture	1 week	Chest pain	CXR, CT	“Dry” epicardial puncture, lead extraction, tined lead implantation
Wang (this article)	F, 78	Screw-in lead	RA free wall	Pericard	No sensing and capture	Immediately	Chest pain, nausea	CXR	Pericardiocentesis, lead extraction, tined lead implantation
Wang (this article)	F, 60	Screw-in lead	RA free wall	Pericard	No sensing and capture, decreased impedance	30 min	Chest pain, nausea, emesis, hemodynamic unstable	TTE, CXR	Pericardiocentesis, lead extraction, new screw-in lead implanted at RA septum

CT, computed tomography; CTPA, CT pulmonary angiography; CXR, chest radiography; RA, right atrium; RAA, right atrial appendage; RCA, right coronary artery; SVC, superior vena cava; TTE, transthoracic echocardiography; M, male; F, female (age in years).

Position denotes where the atrial lead was implanted. Perforation represents the site of the lead tip after the diagnosis of perforation. Pace/sense signifies the type of pacemaker malfunction. Delay is the interval between implantation and the time of perforation. Imaging indicates the positive findings related to atrial lead perforation in imaging examination.

### Manifestation

Lead perforations occurred within and over 1 month of the implantable procedures in 34 (68%) and 16 (32%) patients, respectively. Electrical performance such as capture, sensing, and lead impedance of the atrial lead was not reported in eight patients. Besides 18 patients with no change, the main malfunction was loss of capture in 15 patients, of which 14 were associated with sensing problems, 5 with elevated threshold, and 3 with impedance changes. Therefore, it is important to pay close attention to any discomfort within the first month after pacemaker implantation, even if the changes in pacing parameters are inconspicuous.

A total of 41 patients presented with clinical symptoms, predominantly chest pain in 25 patients. Other concomitant symptoms covered syncope, dyspnea, shortness of breath, hemoptysis, back pain, hiccups, nausea, and emesis. Of these, 12 patients were hemodynamically unstable at the time of detection of the perforations. The perforations were detected in two asymptomatic patients with normal electrical parameters on routine CXR (33, 41). Coronary angiography in one patient with prior coronary artery bypass grafting demonstrated that the atrial lead was protruding into an unusual filling mass communicating with the right coronary artery (RCA) through a fistula (22). In a total of 50 patients, 29 had CT evidence of lead perforation, while CXR illustrated the piercing site in 26 patients with 16 overlapping. Totally nine cases were investigated by TTE alone before intervention.

### Position of the lead tip

In 40 (80%) patients, screw-in leads were perforated, 4 (8%) had passive fixation leads, and the fixation mechanism was unknown in 6 cases. The use of active fixation leads reduces the risk of lead displacement but may increase the risk of perforation. An observational study found that eight patients (42%) experienced subsequent lead displacement, while no patient experienced displacement of an active fixation lead in the coronary sinus for bi-atrial pacing (46). A recent study found that active fixation leads had a considerably reduced rate of displacement within 6 months compared with passive fixation quadripolar leads (1/135, 0.74%, vs. 16/341, 4.69%) for cardiac resynchronization therapy (47). Concerning a total of 40 active fixation leads, 16 were placed in the RA free wall, 9 in the RAA, 2 in the anterolateral RA, and 1 in the anterior RA with 12 not reported. Regarding the 4 cases utilizing tined leads, 1 reported the original lead position in the RAA, while the others were unknown.

In the total cohort of 50 patients, the lead perforated into the pleura in 25 patients, and pericardiac perforations *in situ* occurred in 22 patients. Bleeding from a hole injury in the atrial wall at the site of the RCA was observed in two patients during thoracotomy (31, 43). In another patient, mechanical irritation led to a fistulous connection with the RCA (22). One patient had a hemorrhagic ulcer of the aortic root in the region of the non-coronary sinus caused by friction from the atrial lead (27). In one patient, the dislodged atrial lead exited the superior vena cava (SVC) through a perforation, passed through the dorsal transverse sinus of the ascending aorta, and connected to the roof of the left atrium (28).

### Management

Surgical interventions including thoracotomy, mini-thoracotomy, thoracoscope, and da Vinci robotic system were utilized in 23 patients. Among the rest of the cases, pericardiocentesis was required in seven patients and chest tube insertion in three patients. Repositioning of the initial atrial lead was achieved in 13 patients. In eight patients the protruding lead was removed. For the 13 patients with lead extraction and new lead implantation, 6 were replaced with passive fixation leads, 4 remained with active fixation leads, and 3 had epicardial leads placed. In a patient with hemoptysis, the application of local catecholamines under bronchoscopy decreased bleeding in the right middle lobe (36). Expectant management was continued due to the substantial high risk of lead revision in one patient with underlying severe chronic obstructive pulmonary disease (13) and spontaneous resolution of the pneumothorax in three patients each (30, 33). The postoperative course was uncomplicated in all but one patient (27).

## Discussion

Backtracking along the intraoperative situation, we found that all three patients suffered from breath-dependent chest prickling pain to varying degrees during the procedure. Concerning the readmitted patients with atrial lead perforation, their vital signs were relatively stable. As chest pain should be differentiated from myocardial infarction, aortic dissection, and pulmonary embolism, the diagnosis was established by electrical parameters and imaging examinations. Hemodynamically irrelevant pericardial effusion on TTE should be identified with pericarditis. Significantly, we found that atrial electrode perforation occurring before discharge was generally associated with pericardial effusion and presented with more severe symptoms such as chest distress, spontaneous sweating, and subsequent hemorrhagic shock.

Using screw-in leads, improper fixation strength and excessive attempt times were all closely correlated with the high incidence of cardiac perforation. Furthermore, the influence of the implantation site cannot be ignored. Zoppo et al. assessed the morphology of the RAA in relation to pacing and concluded that the proximal antral RAA is potentially a dangerous region for lead anchorage, primarily because of its proximity to the aortic root and the paper-thin RAA wall in the interpectoral muscular spaces, compared with the safer distal saccular RAA beyond the tenia sagittalis (48). The first patient described validated this conclusion. The thickness of the atrial wall was proportional to the different heights of the RAA isthmuses, as well as the greatest near the valve annulus and the thinnest near the RAA orifice (49). Consistent with literature review, leads implanted in the anterior wall of the RA or the lateral wall of the RAA root have a higher risk of perforation. Based on our experience, if the active lead has been tilted to the aforementioned site while the patient, particularly elderly female patients with a low body mass index (BMI), complains of breath-dependent chest prickling pain during the implantation, the possibility of the lead penetrating the visceral pericardium should be taken into consideration. On this occasion, close attention should be paid to any change in the patient's condition, including symptoms, vital signs, and size of the cardiac silhouette. When there were persistent symptoms or altered electrical parameters, especially notably elevated threshold and decreased impedance, or progressive pericardial effusion, close monitoring following the lead replacement was recommended. Definitely, future studies are needed to elucidate the relationship between atrial electrode perforation and atrial anatomy to further reduce its incidence.

For patients who had hemopericardium and hypotension, urgent pericardiocentesis and fluid resuscitation should be performed. Pericardiocentesis under fluoroscopy was superior to bedside when there was only a minimal volume of pericardial effusion. The observation period before the end of intervention might be appropriately extended. After the hemodynamic stability was obtained, it was time for electrophysiologists to decide how the protruding electrode should be tackled on the basis of electrical parameters and imaging examinations. Patients who do not respond to the aforementioned treatment or who have a cardiac rupture should undergo surgical intervention. Other than ventricular perforation, atrial lead perforation can be safely managed by experienced electrophysiologists in the cath lab.

The “dry” epicardial puncture introduced by Sosa Scanavacca et al. was an established approach for epicardial mapping in patients with ventricular tachycardia after failed endocardial ablation (50). Since then its application has been broadened to ligation of the left atrial appendage, ablation of atrial fibrillation, and implantation of pacemaker leads (51–53). We utilized this method with J-tipped guidewire insertion in the first patient in the event that lead extraction generated hemopericardium. This prophylactic pericardiocentesis was recommended in hemodynamically stable patients with no or merely mild pericardial effusion, especially in patients with heart failure and DCM who could not tolerate the thoracotomy.

Once in-hospital lead perforation was confirmed, right back to the cath lab withdrawing the screw-in lead and replacing with a tined one were recommended. Also, lead repositioning to the atrial septum was generally feasible. Alteration of both the type and position of the atrial lead transvenously was safe and reliable, because in the acute period the fibrous tissue around the protruding electrode has not formed yet, thus its retention could cause further damage to the surrounding tissue and pericardial effusion or tamponade during extraction.

Last but not the least, we should focus on the prevention of the lead perforation, especially when an active fixation lead is arranged to be inserted into the RA free wall in elderly women with low BMI who are quite likely to have thinned out myocardia. The procedure should be gentle and the pacing wire should be reserved of moderate length to avoid excessive tension at the distal tip.

## Conclusions

In summary, atrial lead perforation is likely to occur when an active fixation lead is placed in the RA free wall in elderly patients with low BMI. Our experience is that if the patient has intermittent stabbing chest pain associated with deep breathing during the implantation, atrial perforation should be suspected, which could be confirmed by electrical parameters and imaging examinations. Other than ventricular perforation, the atrial lead perforation in the cath lab can be safely handled by experienced electrophysiologists.

## Data Availability

The original contributions presented in the study are included in the article/Supplementary Material, further inquiries can be directed to the corresponding authors.
